# Obesity, Inflammation, and Prostate Cancer

**DOI:** 10.3390/jcm8020201

**Published:** 2019-02-06

**Authors:** Kazutoshi Fujita, Takuji Hayashi, Makoto Matsushita, Motohide Uemura, Norio Nonomura

**Affiliations:** Department of Urology, Osaka University Graduate School of Medicine, Suita, Osaka 565-0871, Japan; takujihayashi0929@gmail.com (T.H.); matsushita@uro.med.osaka-u.ac.jp (M.M.); uemura@uro.med.osaka-u.ac.jp (M.U.); nono@uro.med.osaka-u.ac.jp (N.N.)

**Keywords:** obesity, inflammation, prostate cancer, immune cells, cytokine, high-fat diet

## Abstract

The prevalence of obesity is increasing in the world, and obesity-induced disease, insulin-resistance, cardiovascular disease, and malignancies are becoming a problem. Epidemiological studies have shown that obesity is associated with advanced prostate cancer and that obese men with prostate cancer have a poorer prognosis. Obesity induces systemic inflammation via several mechanisms. High-fat diet-induced prostate cancer progresses via adipose-secretory cytokines or chemokines. Inflammatory cells play important roles in tumor progression. A high-fat diet or obesity changes the local profile of immune cells, such as myeloid-derived suppressor cells and macrophages, in prostate cancer. Tumor-associated neutrophils, B cells, and complements may promote prostate cancer in the background of obesity. Interventions to control systemic and/or local inflammation and changes in lifestyle may also be viable therapies for prostate cancer.

## 1. Introduction

Since 1980, the prevalence of obesity has doubled in the world. Obesity is caused by genetic factors, neuroendocrine factors, psychological factors, and environmental factors [1]. In the United States, almost 40% of people suffer from obesity, and the present situation is a “pandemic” of obesity. In Asian countries such as Korea and Japan, the prevalence of obesity is still low, approximately less than 10%, but the prevalence of obesity has increased over the last decade [1,2]. The incidence rate of prostate cancer is also increasing and is now highest in Japan. Overfeeding with a high-fat and/or high-calorie diet and less physical activity result in an energy imbalance and adiposity. Obesity causes insulin resistance, type 2 diabetes, cardiovascular diseases, and several malignancies via systemic inflammation. The resulting medical costs due to obesity are increasing and becoming an important issue worldwide.

**Figure 1:**
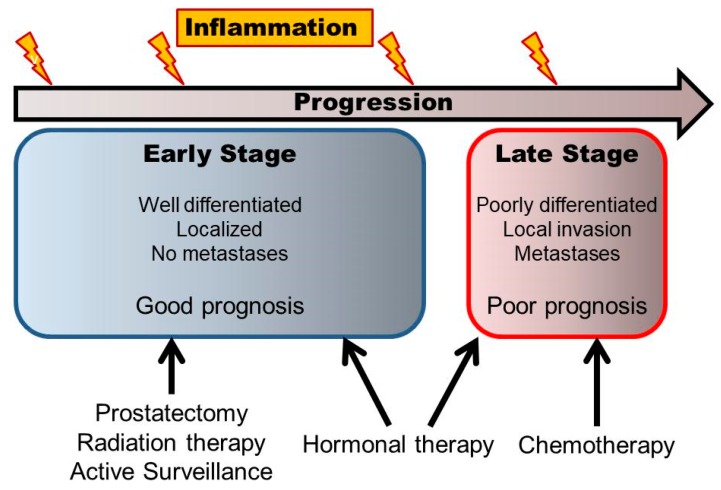
The scheme of different stages and progression of prostate cancer.

Prostate cancer has had high morbidity among elderly men. Many patients with prostate cancer are in the early stage and have good prognosis after several treatments including prostatectomy, radiation therapy, hormonal therapy, and even active surveillance. However, some progressive prostate cancer patients in the late stage with poorly-differentiated cancer cells, or local invasion, or metastatic lesion are more resistant to several treatments including hormonal therapy or chemotherapy, and have poor prognosis. (Figure 1) It is important to elucidate the mechanism of the factors inducing prostate cancer progression.

Chronic inflammation is the major etiology behind the development of several cancers, such as hepatocellular carcinoma, squamous cell carcinoma in the urinary bladder, colorectal cancer, and gastric cancer. Inflammatory cells migrating to the local area generate reactive oxygen species and reactive nitrogen species that induce mutations of DNA in normal epithelia [3]. Acute or chronic inflammation is a common histological finding in both benign and malignant tissues in prostatectomy specimens [4,5]. The causes of inflammation in the prostate vary among bacteria causing prostatitis and sexually-transmitted disease, hormonal changes of estrogen [6], physical trauma caused by corpora amylacea [7,8], urine reflux to the prostate gland, and environmental factors such as dietary habits [9,10]. Dietary habits cause inflammation of the prostate and can result in carcinogenesis in the early stage [11,12]. Dietary-induced inflammation could last for the entire life, and chronic inflammation can also stimulate the progression of prostate cancer in the late stage. However, the association of immune cells in tumor microenvironments with prostate cancer is still unclear.

In this review, the link between obesity and prostate cancer is discussed based on the recent findings related to inflammation.

## 2. Obesity and Prostate Cancer

Several studies reported that obesity was associated with the increased risk of several cancers, such as colon, breast, endometrial, kidney, gastric, esophagus, pancreas, liver, and gall bladder [13,14]. Several studies have shown the association of obesity with the risk of prostate cancer. A prospective study of 3673 men in the United States showed that greater body mass index (BMI) was an independent predictor of prostate cancer (relative risk = 1.7 for BMI > 27.8 kg/m^2^ compared with <23.6 kg/m^2^; *p* = 0.1). The percent change in BMI from baseline to age 50 was also positively associated with risk (*p* = 0.01) [15]. Another prospective study in the United States showed that BMI was weakly and positively associated with prostate cancer, and the association of obesity with the risk of clinically-significant prostate cancer strengthened after the exclusion of well-differentiated, localized tumors [16]. However, a prospective study of 36,959 Swedish men showed that the incidence of localized prostate cancer was inversely associated with BMI in middle-to-late adulthood (the rate ratio for 35 kg/m^2^ when compared with 22 kg/m^2^ was 0.69 (95% confidence interval (CI) 0.52–0.92)), but not in early adulthood. BMI in middle-to-later adulthood was associated with a non-statistically significant increase in the risk of fatal prostate cancer (rate ratio for every five-unit increase: 1.12 (0.88–1.43)) and BMI in early adulthood with a decreased risk of fatal prostate cancer (rate ratio for every five-unit increase: 0.72 (0.51–1.01)) [17]. A prospective study of 141,896 men in the European Prospective Investigation into Cancer and Nutrition (EPIC) cohort showed that high BMI at a young age was inversely associated with the overall risk of prostate cancer (relative risk = 0.89, 95% CI 0.80–0.98, BMI ≥ 26 vs. 20–21.9, *p* = 0.01) and with fatal and advanced disease [18]. Obesity at a young age causes the delayed onset of puberty and may result in the lower lifetime exposure of insulin-like growth factor 1 (IGF-I), which may affect the development of prostate cancer later in life [18,19]. A meta-analysis of 12 prospective studies of localized prostate cancer (1,033,009 men, 19,130 cases) and 13 of advanced prostate cancer (1,080,790 men, 7067 cases) showed an inverse linear relationship with BMI for localized prostate cancer (*p* ≤ 0.001, relative risk: 0.94 for every 5-kg/m^2^ increase) and a positive linear relationship with BMI for advanced prostate cancer (*p* = 0.001, relative risk: 1.09 for every 5-kg/m^2^ increase) [20]. Obesity thus could affect the incidence of the risk of prostate cancer in the early stage in the opposite direction according to the type of prostate cancer. The underlying mechanisms of this inverse association of obesity with localized prostate cancer could be the low testosterone levels in obese men. Obese men have a lower concentration of free testosterone due to a decrease of lutenizing hormone (LH) pulse amplitude and serum LH levels [21]. Plasma total testosterone and free testosterone were positively associated with increased risk of low-grade prostate cancer [22]. However, the association of testosterone, free testosterone, and the free-to-total testosterone ratio with prostate cancer is still controversial [23]. Furthermore, the impact of obesity-induced systemic inflammation on the inverse relationship of localized prostate cancer to BMI is still unknown.

Obesity may also affect the prognosis of prostate cancer in the late stage. An analysis of 4123 men treated by radical prostatectomy showed that higher BMI was associated with biochemical recurrence after radical prostatectomy (hazard ratio (HR) 1.02, 95% CI 1.00–1.02, *p* = 0.008) [24]. A retrospective analysis of 4268 radical prostatectomy patients within the Shared Equal Access Regional Cancer Hospital (SEARCH) database showed that being overweight and obesity were associated with prostate cancer-specific mortality (HR 1.88, *p* = 0.061 and HR 2.05, *p* = 0.039, respectively) [25]. A prospective study of 404,576 men showed a positive linear trend in the prostate cancer death rate with higher BMI (*p* < 0.001) [14]. These epidemiological studies showed obvious evidence of the association of obesity with advance prostate cancer.

## 3. Obesity and Inflammation

Many studies have shown that obesity causes systemic inflammation through the action of various mechanisms. Adipocytes secrete tumor necrosis factor (TNF)-α in obese mice that causes systemic inflammation [26]. A high-fat diet (HFD) changes the intestinal microbiota and increases the translocation of live Gram-negative bacteria through the intestinal mucosa into the bloodstream and mesenteric adipose tissue, which results in continuous bacteremia [27]. Fatty acids activate toll like receptor 4 (TLR4) signaling in adipocytes and macrophages. Female mice lacking TLR4 show increased obesity, but are partially protected against HFD-induced insulin resistance, possibly due to reduced inflammatory gene expression in the liver and fat [28]. Obesity induces activation of the innate immune system. Adipose depots contain multiple immune cells. Macrophages in adipose tissues are increased in the obese, skewing to the M1-polalized macrophages. These macrophages show a pro-inflammatory phenotype and secrete inflammatory cytokines such as TNF-α [29].

**Figure 2:**
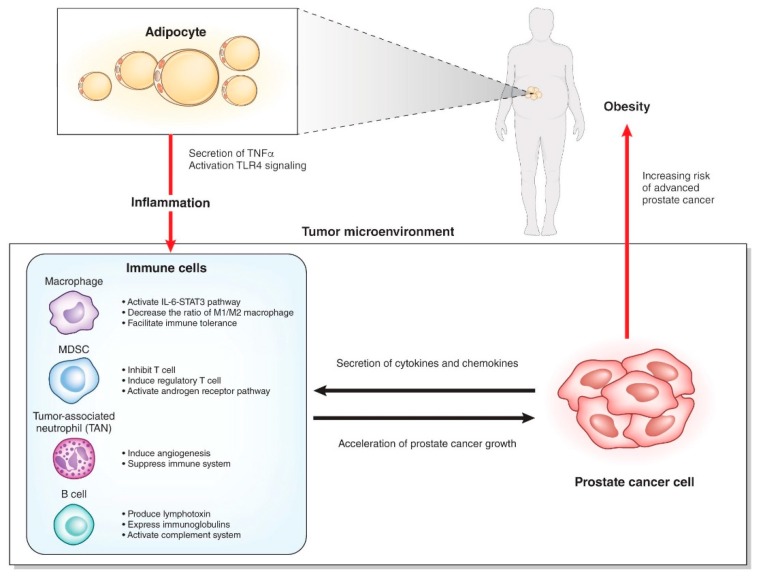
Interaction of immune cells with adipocytes and prostate cancer cells.

It is still unclear how such systemic inflammation affects local inflammation of the prostate (Figure 2). Several chemokines and cytokines secreted from prostate cancer cells may recruit immune cells to the prostate. Which organ are these immune cells activated in? Some immune cells could be “taught” in the intestinal wall [30], but there has been no evidence of the homing of these intestinal immune cells to a distant organ. In bone marrow or regional lymph nodes, the immune cells might be activated by factors related to obesity and subsequently recruited to the prostate. Otherwise, the local immune cells recruited by prostate cancer cells might be activated by the obesity-related factors. The elucidation of these factors related to obesity could lead to the development of new treatments or the prevention of prostate cancer in the early stage.

## 4. Obesity Promotes Prostate Cancer Growth

Although the link between obesity and prostate cancer has not been definitively determined, several studies focusing on the cytokines and/or chemokines have been reported. In a mouse xenograft model of the prostate cancer cell line LNCaP, serum monocyte chemoattractant protein-1 (MCP-1) was significantly increased, and tumor growth was promoted in HFD-fed mice [31]. Palmitic acid is one of the saturated free fatty acids abundantly included in HFDs. The addition of palmitic acid induced the expression of macrophage inhibitory cytokine 1 (MIC1) in vitro, and serum levels of MIC1 were increased in the HFD-fed mice xenograft model. Obese patients with prostate cancer were also found to have higher serum levels of MIC1 than those in healthy controls [32]. HFDs also modulate miRNA expression in prostate cancer cells. Prostate cancer cells cultured in the serum of HFD-fed mice showed a marked increase in cell proliferation and the attenuation of miR-130a. miR-130a modulated MET expression in prostate cancer cell lines, and furthermore, cytoplasmic MET in prostate cancer tissues was overexpressed in patients with higher BMI [33]. An HFD also induced increases in leptin, C-C motif ligand (CCL)3, CCL4, CCL5, and C-X-C motif ligand (CXCL)10 in the sera of transgenic adenocarcinoma of mouse prostate (TRAMP) mice. The conditioned medium of sera from HFD-fed TRAMP mice promoted the proliferation, migration, and invasion of DU-145 cells [34]. Obese patients with prostate cancer showed increased expression of epithelial CXCL1, which induces the recruitment of adipose stromal cells from white adipose tissue to the tumor and promotes the tumor’s growth [35]. These reports showed that cytokines and chemokines could play important roles in the obesity-associated progression of prostate cancer in the early and late stage. Because TRAMP mice lacking expression of androgen receptor are thought to be models for a very advanced stage with neuroendocrine cancer cells and independent from androgen receptor, the findings using TRAMP mice might be compatible with prostate cancer patients in only the late stage. Moreover, the detailed mechanisms including the tumor microenvironments are still unknown.

## 5. Inflammation in Prostate Cancer

In the tumor microenvironments, the interactions among cancer cells, immune cells, endothelial cells, and fibroblasts can play important roles. Inflammatory cells consist of innate immune cells and acquired immune cells. Acquired immune cells include B cells and T cells, which act based on antigen recognition. While innate immune cells are the main players in inflammation, innate immune cells and acquired immune cells also orchestrate the inflammation. Innate immune cells including neutrophils, myeloid cells, mast cells, and macrophages are different from acquired immune cells by receptor-mediated activation and their rapid response to invading pathogens and foreign bodies [36]. Macrophages and neutrophils are the most abundant immune cells in the tumor microenvironment [37].

**Figure 3:**
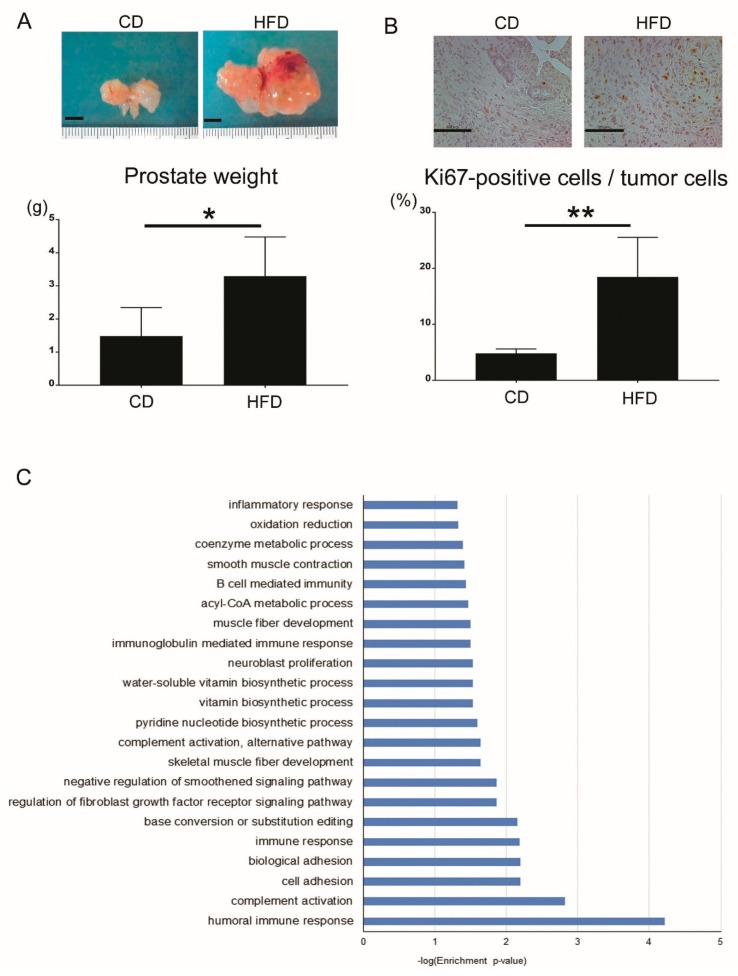
(**A**) Representative gross findings of the prostatic tissues (the black bar indicates 5 mm) (top) and prostate weights (*n* = 6 and 4, respectively) (bottom) of the model mice at 22 weeks of age. (**B**) Representative images of Ki67 staining for the prostatic tissues (top) and the ratio of Ki67-positive cells to tumor cells (*n* = 4 and 3, respectively) (bottom) of the model mice at 22 weeks of age. (**C**) Gene ontology analysis using mRNA microarray technology of the prostatic tissues of the model mice at 22 weeks of age (HFD-fed vs. CD-fed, *n* = 3, respectively; fold change >2.0, *p* < 0.05, biological process). CD, control diet; HFD, high-fat diet. * *p* < 0.05, ** *p* < 0.01.

To reveal the relationship between HFD-induced inflammation and tumor progression in the prostate, we used two genetically-engineered prostate cancer mouse models, prostate-specific *Pten* knockout mice (Pb-Cre^+^; *Pten*(fl/fl)) and *Pten* and *Tp53*-double knockout mice (Pb-Cre^+^; *Pten*(fl/fl); *Tp53*(fl/fl)) on the C57BL/6 genetic background. The prostate weights and the ratio of Ki67-positive cells to tumor cells, which indicates the proliferative capacity of the tumor, of the mice in the HFD-fed double knockout mouse model were significantly higher than those of the control diet (CD)-fed model mice (*p* = 0.011, *p* = 0.005, respectively) (Figure 3A,B). Total RNA was isolated from prostatic tissues of both the CD-fed mice and HFD-fed double knockout mice, and transcriptome analysis of the two groups was performed using mRNA microarray technology. Gene ontology analysis revealed that many processes related to inflammation and the immune response were ranked in the top 22 processes expressed in the prostate of the HFD-fed double knockout mice (Figure 3C). This finding strongly suggests that local inflammation of the prostate is one of the most important factors for the progression of prostate cancer in obese or HFD-fed mice in the early and late stages. The profiles of the local immune cells in prostate cancer were analyzed in the *Pten* knockout mouse model fed with a CD or HFD. Although the number of B cells, T cells, macrophages, and mast cells and the ratio of CD8/CD4 T cells were not changed by the HFD, the number of myeloid-derived suppressor cells (MDSCs) and the M2/M1 macrophage ratio were significantly increased in the HFD-fed mice compared with the CD-fed mice. The promotion of tumor growth by the HFD was completely cancelled by the administration of celecoxib, a cyclooxygenase 2 (COX-2) inhibitor, which suggests that inflammation plays a central role in tumor progression caused by an HFD. IL-6 expression in prostate tissues was increased in HFD-fed mice, as were the amounts of phosphorylated signal transducer and activator of transcription 3 (STAT3) in prostate cancer cells. Inhibition of the IL-6 pathway resulted in the suppression of tumor growth by an HFD [38]. The HFD and subsequent obesity caused the increased secretion of IL-6 from local macrophages in the prostate tumor via unclear mechanisms. IL-6 might increase the number of local MDSCs and promote the proliferation of prostate cancer cells via signal transducer and activator of transcription 3 (STAT3) pathways. Because transcriptome analysis in double knockout mice resulted in different changes of gene expressions from *Pten* knockout mice after administration of HFD, *Tp53* may have many functions regarding inflammation. In addition, it might result in different findings if model mice on the other genetic background were to be examined.

## 6. Macrophages

Macrophages, one of the most abundant types of immune cells in tumor microenvironments, change the phenotype to promote tumor growth and metastasis. Macrophages are divided into classic macrophages (M1) and alternative macrophages (M2). M1 macrophages act in microbicidal and tumoricidal activity, and M2 macrophages act in tissue remodeling, immune tolerance, and tumor progression [39]. M1 macrophages are characterized by the secretion of interleukin-1β (IL-1β), IL-6, IL-12, and TNF-α, whereas M2 macrophages are characterized by the secretion of IL-4, IL-10, and TGF-β. At early stages of tumor development, macrophages undergo classic activation and exhibit an M1 phenotype [36,40]. Cytokines secreted from M1 macrophages play roles in tumor initiation and early promotion [36]. Exposure of macrophages to IL-4, colony-stimulating factor-1 (CSF1), granulocyte-macrophage colony-stimulating factor (GM-CSF), and TGFβ secreted by cancer cells polarize macrophages to the M2 phenotype, which acts to induce immunosuppressive microenvironments. Inflammatory cytokines secreted from adipocytes, such as TNF-α, IL-6, IL1β, and CCL2, recruit macrophages to the adipose tissues. Diet-induced obesity leads to a shift of the macrophage phenotype from M2 to M1 in mice [41,42]. In contrast, in mammary adipose tissue of breast cancer in obese women, macrophages showed a decrease in the expression of IL-10 and CD11c, which are characteristic of an M1 polarization phenotype. However, they also showed an increase in the expression of CD206, which is a surface marker of the M2 polarization phenotype, suggesting a mixed polarization phenotype in tumor microenvironments [39,43]. Macrophages are known to promote cancer growth and metastasis in prostate cancer, but the association of macrophages with obesity in prostate cancer is still unclear. Different from breast cancer, adipocytes are located in the area surrounding the prostate and are not found within the prostate tissues. Prostate cancer and stromal cells secrete CCL2, which strongly recruits macrophages [44,45]. CCL2 levels were increased in the sera of HFD-fed mice with an LNCaP xenograft. It is also reported that the number of tumor-infiltrating macrophages is not associated with BMI [46]. The role of macrophages in prostate cancer with a background of obesity will require further study.

## 7. Myeloid-Derived Suppressor Cells (MDSCs)

MDSCs have a strong immunosuppressive function that enables the regulation of immune response and suppresses overt inflammatory responses [47]. MDSCs represent a non-lymphoid immune suppressor cell population of myeloid origin that is enriched in cancer [48]. MDSCs are a heterogeneous population and express a mixture of surface markers typical for myeloid cells, but they lack the markers of lymphocytes, natural killer cells, macrophages, and dendritic cells [47]. MDSCs were originally found in mice, and their counterparts in humans are not well defined. MDSCs in mice are characterized by the surface marker Gr-1+CD11b+. MDSCs are divided into two major groups: cells with a morphology and surface markers characteristic of monocytes (monocytic (M)-MDSCs) and cells with surface markers characteristic of polymorphonuclear (PMN)-MDSCs). In mice, M-MDSCs are characterized by the surface markers of CD11b^+^Ly6C^high^ Ly6G^–^, and PMN-MDSCs are characterized by CD11b^+^Ly6C^low^ Ly6G^+^. In humans, the equivalent cells to PMN-MDSCs are defined as CD11b^+^CD14^–^CD15^+^ or CD11b^+^CD14^–^CD66b^+^ and M-MDSCs as CD11b^+^CD14^+^HLA-DR^–/low^ CD15 [49]. MDSCs are characterized by the suppression of T cell response by ARG1, iNOS, and reactive oxygen species. MDSCs inhibit T cells via arginase-1, iNOS, and ROS and induce regulatory T cells by IL-10 and TGF-β. MDSCs also modulate the cytokine production of macrophages and promote tumor angiogenesis and eventually metastasis [47]. In a prostate cancer mouse model (TRAMP mouse), IL-23 secreted from MDSCs can activate the androgen receptor pathway and promote cell survival and proliferation under an androgen-deprived condition. Blockade of IL-23 can oppose MDSC-mediated resistance to castration in prostate cancer [50]. CXCL5 secreted from prostate cancer cells attracts MDSCs expressing CXCR2 in a mouse model of prostate cancer. Elimination of MDSCs or the blocking of CXCL5-CXCR2 signaling elicits an antitumor response for prostate cancer [51]. In humans, CD14+HLA-DR^–/low^ M-MDSCs and Treg were significantly increased in peripheral blood from patients with prostate cancer compared with healthy donors. High levels of M-MDSCs in the blood were associated with a shorter median overall survival [52]. In patients with prostate cancer, MDSCs accumulate in the blood as prostate cancer progresses and inhibit the proliferation of autologous CD8+ T cells and the production of interferon-γ (IFN-γ) and granzyme-B [53]. MDSCs could be a new target in the prevention and treatment of prostate cancer and/or castration-resistant prostate cancer.

## 8. Neutrophils

Neutrophils primarily work as an antibacterial immune response, but tumor-associated neutrophils (TANs) also play important roles in tumor microenvironments. Similar to M1 and M2 macrophages, terms for antitumoral N1 neutrophils and protumoral N2 neutrophils were proposed [54]. The chemokines CXCL1, CXCL2, CXCL5, CXCL6, and CXCL8 secreted from tumor cells attract neutrophils in the blood to the tumor microenvironment via CXCR1 and CXCR2 on the surface of neutrophils [55]. TANs share a similar surface marker with PMN-MDSCs. Murine neutrophils are defined as CD11b+/GR1+/Ly6G+cells, whereas PMN-MDSCs are defined as CD11b^+^/GR1^high^/Ly6G^+^ cells. PMN-MDSCs were named based on the function of immunosuppression. However, neutrophils can work in immunosuppression, but also have the opposite function of anti-tumor activity. N1 TANs function in tumor cell cytotoxicity, CD8+ T cell recruitment, and antibody-dependent cell-mediated cytotoxicity. In contrast, N2 TANs play roles in angiogenesis, immunosuppression, and tumor growth via several cytokines or proteins released from TANs [55]. In a mouse model, obesity caused the increase of neutrophils in the lung and promoted the metastasis of breast cancer cells to the lung in a GM-CSF- and IL-5-dependent manner [56]. In HFD-fed mice, cholesterol metabolites promoted the metastasis of breast cancer via neutrophils and γδ-T cells [57]. In another mouse model, obesity promoted the progression of pancreatic cancer and resistance to chemotherapy via TANs recruited by adipocyte-secreted IL1β [58]. Murine neutrophils are different from human neutrophils. Thus, it is still unclear whether the TANs play roles in prostate cancer progression in the late stage. The administration of cabozantinib resulted in the clearance of prostate cancer in mice by recruiting neutrophils to the tumor [59].

In humans, no markers equivalent to the mouse Gr1 marker exist, and human neutrophils are defined as CD14-/CD15^+^/CD66b^+^/CD16^+^. The neutrophil-lymphocyte ratio in peripheral blood is associated with a high Gleason score and the poor prognosis of men with prostate cancer [60,61]. The ratio is also a prognostic factor of abiraterone and docetaxel treatment in men with castration-resistant prostate cancer [62,63]. Low serum neutrophil count is a predictor of positive prostate biopsy results [64]. The presence of neutrophils in the epithelial lining of the prostate gland indicate prostatic inflammation and is a predictive factor of benign biopsy [65]. The protumor roles of neutrophils in human prostate cancer have not been confirmed yet, and further studies are warranted.

## 9. B Cells and Complements

In the mRNA microarray analysis of prostate tumors in CD- and HFD-fed double knockout mice, the expressions of immune-related genes including splice variants of immunoglobulins, complements (*Hc*, *C4b*), *Ccl8*, and *Cd52* were significantly higher in HFD-fed double knockout model mice compared with CD-fed double knockout model mice (Table 1). Gene ontology analysis revealed that humoral immune responses were key factors of HFD-induced tumor progression (Figure 3C). These results suggested that B cell-mediated and immunoglobulin-mediated immune responses could be key factors of HFD-induced prostate cancer growth. B cells play important roles in diet-induced obesity, chronic inflammation, and humoral immunity, the latter two of which are influenced by some kinds of fatty acids and lipid mediators [66]. B cells are also related with tumor progression in various types of cancer, including prostate cancer [67]. Tumor-infiltrating B cells produce lymphotoxin, a cytokine belonging to the TNF family, that leads to activation of IκB kinase α and STAT3, which promote the survival and proliferation of androgen-deprived prostate cancer cells that result in the development of a castration-resistant state in experiments using the TRAMP mice model [68]. It was reported that higher B cell infiltration was present within the intra-tumoral prostate cancer regions compared to the extra-tumoral benign prostate tissue regions in prostatectomy sections [69]. Immunoglobulins are expressed by B cells and a variety of tumor tissues and cancer cell lines [70,71]. Immunoglobulins are suggested to play important roles in promoting cancer progression. Immunoglobulin G silencing induced apoptosis and suppressed proliferation, migration, and invasion in LNCaP prostate cancer cells [72].

A complement system is also related to cancer progression [73,74]. Complement activation in the tumor microenvironment enhances tumor growth and increases metastasis. The hemolytic complement encoded by the *Hc* gene in mice, the expressions of which were increased in the prostatic tissues of HFD-fed mice in our results, corresponds to C5 in human. C5, one of the complements, was suggested to promote tumor progression controlling the tumor microenvironment [75,76]. A humoral immune response including B cells, immunoglobulins, and complements could be key factors of prostate cancer progression induced by inflammation in the late stage. The detailed mechanism of the phenomenon remains unclear, and further investigations are necessary to explore the causative mechanism.

## 10. Conclusions

Inflammation and immune responses play important roles in the progression of prostate cancer. Other inflammatory cells and immune cells could be also involved in the prostate cancer progression. T cells are also accumulated in prostate cancer of a diet-induced obese Hi-Myc mice [77]. The cytotoxic function of NK cells to prostate cancer cells is inhibited by humoral factors from adipocytes [78]. These local inflammatory cells are orchestrated by several signalings from immune cells, adipocytes, or prostate cancers. Prostate cancer cells stimulated by adipokines or saturated fatty acid could change the local immune profile in the backgrounds of obesity [79]. The interplay between prostate cancer and immune cells is a “chicken and egg” situation. Another possible mechanism to affect prostate cancer in obesity could be an intestinal microbiome. High-fat diet changes the intestinal microbiome and enhances colorectal cancer and liver cancer [80,81]. The microbiome could modulate the host immune system, and these changes in the immune system might have an effect on distant prostate cancer. Murine immune systems are different from human, and all the findings in mice model could not be extrapolated to human prostate cancer. However, common mechanisms would exist also in human prostate cancer. Further analysis in mice model would give new insights into the mechanisms of the progression of prostate cancer enhanced by obesity and inflammation. Interventions to address systemic and/or local inflammation and a change in lifestyle may be therapeutic for prostate cancer.

## Figures and Tables

**Table 1:** The list of the gene symbols that were highly expressed in the prostatic tissues in HFD-fed mice compared to CD-fed mice (fold-change >2.0, *p* < 0.05, mRNA microarray).

Gene Symbol	Fold-Change (HFD-fed vs. CD-fed)	*p*-Value (HFD-fed vs. CD-fed)
LOC238440	17.739	0.0163
Ighv6-6	12.429	0.0170
Ighv3-8	10.111	0.0048
Ighv14-3	9.678	0.0395
Igkv10-96	8.240	0.0248
Mug1	5.773	0.0068
Snord13	5.003	0.0002
Igkv10-94	4.652	0.0260
Adck1	4.644	0.0261
Itm2a	4.559	0.0223
Igh-VJ558	4.509	0.0371
Gm830	4.480	0.0304
Igkv10-95	4.070	0.0303
Igj	4.029	0.0152
Igh-V3660	3.978	0.0244
Ighj4	3.787	0.0016
Igh-VJ558	3.765	0.0035
Igkv4-55	3.733	0.0171
Igkv4-59	3.716	0.0132
Igkv16-104	3.596	0.0194
Igkv4-91	3.542	0.0456
Ccl8	3.450	0.0002
Ighv1-76	3.350	0.0055
Slc17a4	3.333	0.0124
LOC637260	3.242	0.0237
Hc	3.179	0.0337
Tm4sf4	3.040	0.0424
Ighv1-42	3.018	0.0333
Igkv5-45	3.005	0.0095
Ighv14-4	3.003	0.0255
Ighv1-80	2.997	0.0095
Igkv4-72	2.952	0.0091
Ms4a12	2.946	0.0289
A1cf	2.911	0.0180
Ighv1-77	2.876	0.0240
Adamts5	2.800	0.0341
Gm13307	2.793	0.0064
Clstn2	2.784	0.0280
Igkj5	2.766	0.0091
Ighv5-17	2.765	0.0324
Pdlim3	2.763	0.0099
Ighj3	2.728	0.0045
Myh11	2.709	0.0351
Ighm	2.708	0.0376
Tcrg-V4	2.676	0.0445
Svep1	2.673	0.0410
Ighj1	2.651	0.0290
Iglv1	2.632	0.0041
Pcp4	2.626	0.0476
Cpxm2	2.617	0.0333
Maob	2.616	0.0196
Igkv4-70	2.596	0.0396
Pgm5	2.594	0.0453
Cyp2c68	2.583	0.0168
Igkv4-53	2.582	0.0451
Ighv1-62-2	2.526	0.0011
Ppef1	2.516	0.0118
Acnat1	2.511	0.0460
Gm13304	2.500	0.0403
Igkv12-89	2.463	0.0495
Igh-VX24	2.457	0.0235
Snord14e	2.437	0.0266
Gm13304	2.413	0.0414
Thbs4	2.352	0.0425
Mylk	2.352	0.0185
Cd52	2.345	0.0066
Abca8a	2.313	0.0066
Kcnab2	2.291	0.0122
Inmt	2.290	0.0449
Igh-V3660	2.272	0.0399
Igsf23	2.260	0.0138
Cd200	2.250	0.0255
Dkk2	2.236	0.0418
Acta1	2.225	0.0401
Hhip	2.212	0.0093
Ecm2	2.208	0.0109
Lgi2	2.180	0.0343
Igkv4-62	2.167	0.0373
Prelp	2.151	0.0243
Igkj1	2.143	0.0113
Nlrp6	2.112	0.0417
Gm5485	2.062	0.0280
Serpini1	2.016	0.0088
LOC102642448	2.014	0.0095
Kmo	2.009	0.0386
C4b	2.009	0.0254
Igkv4-53	2.001	0.0105
